# Abducens Nerve Paralysis Induced by a Primary Solitary Sphenoid Sinus Mucocele with Broad Osseous Thinning at the Cranial Base

**DOI:** 10.1155/2020/8897868

**Published:** 2020-09-30

**Authors:** Shoji Naito, Hidenori Yokoi, Yuma Matsumoto, Michitsugu Kawada, Kohei Inomata, Masachika Fujiwara, Arisa Ohara, Koichiro Saito

**Affiliations:** ^1^Department of Otolaryngology-Head and Neck Surgery, Kyorin University School of Medicine, Tokyo 181-8611, Japan; ^2^Department of Pathology, Kyorin University School of Medicine, Tokyo 181-8611, Japan; ^3^Department of Radiology, Kyorin University School of Medicine, Tokyo 181-8611, Japan

## Abstract

Primary solitary sphenoid sinus mucocele is rare, generally presenting with headaches or eye symptoms at the anatomical site. We report the case of a 39-year-old woman incidentally diagnosed with sphenoid sinus mucocele during a complete medical checkup. Imaging revealed that the cystic wall had developed from the rear sphenoid sinus and had spread expansively to diminish the clivus; however, no symptoms were reported, and the patient was managed with close observation. During the follow-up period, diplopia developed suddenly due to isolated left-sided abducens nerve paralysis. An endoscopic endonasal approach was used to open the frontal cystic wall, and fascia lata and fat were used for cranial base reinforcement to avoid future cerebrospinal fluid leakage, resulting in improvement during the early stages of follow-up. Treatment options for sphenoid sinus mucoceles include close observation or surgery. In our case, we chose surgery because of an acute symptomatic manifestation during observation.

## 1. Introduction

Paranasal sinus mucoceles are encapsulated benign mucoceles with an expansive and locally pressing nature. They occur in the paranasal cavity and are filled with mucus and covered by epithelium [1, 2]. These lesions can be divided into two types. Primary mucoceles develop from a variety of underlying abnormalities, including inflammatory mucus drainage obstruction, secretory obstruction, cystic expansion of a mucous gland, and cystic degeneration of polyps. Secondary mucoceles are induced by mucus drainage obstruction resulting from trauma or surgery [3]. While paranasal sinus mucoceles can occur anywhere in the paranasal cavity, a primary or solitary occurrence in the sphenoid sinus is rare [4]. The slow expansion of mucoceles can result in thinning of the adjacent bone and impingement on adjacent vital structures [5].

The diagnosis of sphenoid sinus mucocele is often delayed because these lesions typically do not induce epistaxis and are located deep in the rear nasal cavity, rarely causing symptoms of nasal obstruction. Various symptoms eventually emerge, however, based on the lesion's growth pattern, as the cystic wall can be adjacent to the internal carotid artery, optic nerve, and cavernous sinus [4]. Visual disturbance is the most common ocular symptom [6], followed by motility disorders [7] affecting the oculomotor nerve. Abducens or trochlear nerve involvement occurs less frequently [7]. Here, we report the clinical course of a patient with a primary sphenoid sinus mucocele incidentally diagnosed during a complete medical checkup, with development of an isolated abducens nerve paralysis during the observation period.

## 2. Case Presentation

Our patient provided written informed consent for the publication of all clinical information.

A 39-year-old woman was referred to our department after being diagnosed with sphenoid sinus lesions by magnetic resonance imaging (MRI) performed during a complete medical checkup of the brain. The patient did not report any symptoms during the initial evaluation and showed no evidence of a lump or polyplike lesion in the nasal cavity during the physical examination.

**Figure 1:**
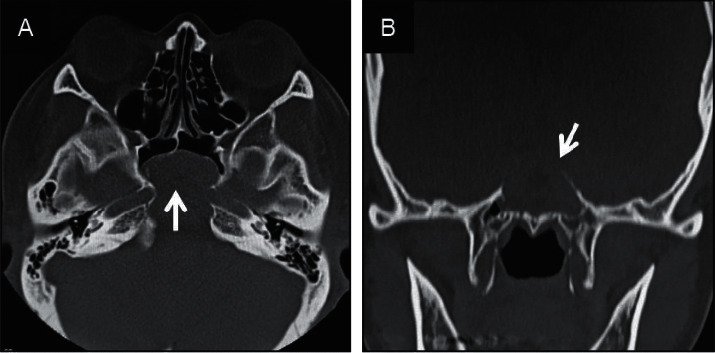
Noncontrast computed tomography imaging of the paranasal cavity at the initial diagnosis. (a) Axial and (b) coronal views. We confirmed the presence of soft tissue contrast, suggesting a retention cyst at the rear sphenoid sinus, with thinning at the sella turcica and left internal carotid artery and elimination of the clivus (white arrows).

**Figure 2:**
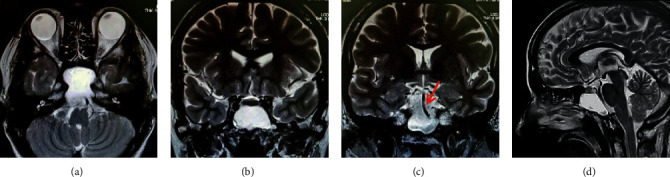
Noncontrast magnetic resonance T2-weighted imaging of the paranasal cavity at the initial diagnosis. (a) Axial, (b, c) coronal, and (d) sagittal views. The basilar artery located adjacent to the cyst (c) is indicated with the red arrow, and the cyst located adjacent to the brainstem (d) is indicated with the white arrow; however, the signal intensity of the lesion's contents is determined to be different from that of cerebrospinal fluid, therefore suggesting a primary cyst.

The presence of an expansive lump, likely originating from the rear sphenoid sinus, was confirmed on noncontrasted computed tomography (CT) (Figure 1). We also identified thinning at the base of the sella turcica and the left internal carotid artery, with the clivus entirely diminished. The lump edges were smooth with a uniform internal density, showing a higher density than that of cerebrospinal fluid. Additionally, the presence of a space-occupying lesion at the same location with compression and an expansive growth pattern was confirmed by MRI (Figure 2). The mucocele was adjacent to the basilar artery secondary to clivus loss, with uniformly dense contents showing a low signal intensity on T1-weighted images and high signal intensity on T2-weighted images, which was a different pattern than that observed for cerebrospinal fluid. Therefore, the patient was diagnosed with sphenoid sinus mucocele. The patient denied a history of paranasal sinus surgery or head trauma, indicating a primary lesion.

**Figure 3:**
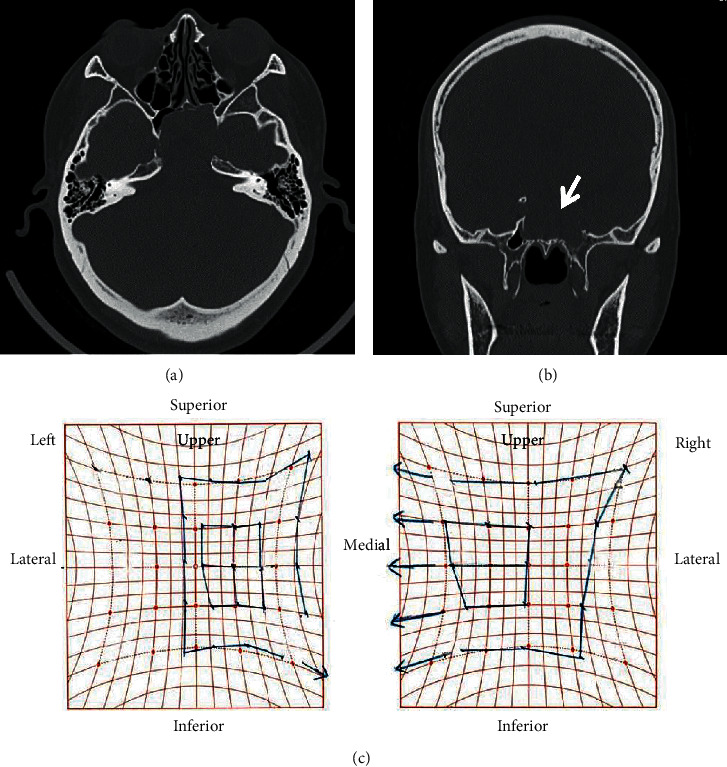
Noncontrast computed tomography imaging of the paranasal cavity and Hess screen test results at the emergency reexamination. CT images with (a) axial and (b) coronal views. The sphenoid sinus mucocele shows apparent growth, with increased impingement on the left superior orbital fissure (white arrow). (c) The Hess screen test confirms a disturbance in the left eye abduction and overaction in the right eye adduction.

We chose nonsurgical treatment because the patient was asymptomatic and reluctant to undergo surgery. Therefore, we implemented regular follow-up visits and CT scans every 6 months. Subsequently, we did not observe any disease progression during the two clinical follow-ups. Fifteen months after the initial diagnosis, the patient experienced acute diplopia upon waking one morning and immediately visited our department for an emergency reexamination (Figure 3). At this time, CT and MRI findings of the paranasal cavity showed growth of the cystic lesion with compression of the superior orbital fissure. The patient was subsequently diagnosed with left abducens nerve paralysis by the Department of Ophthalmology.

**Figure 4:**
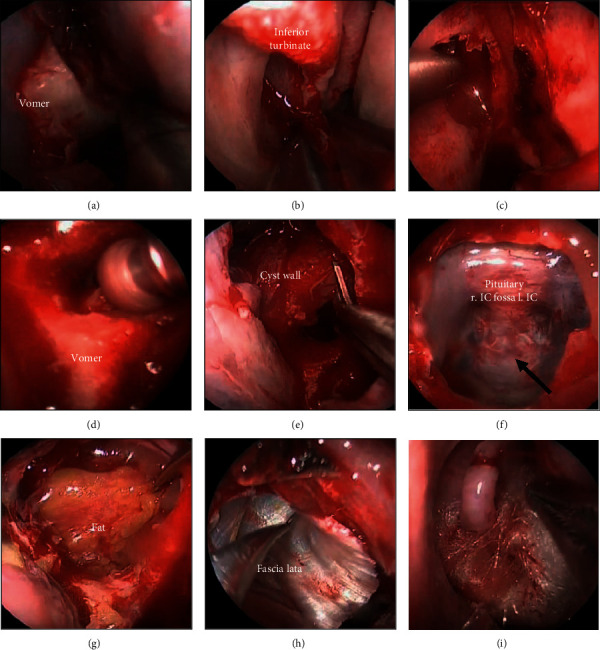
Surgical findings. (a) Remedial surgery is performed on the nasal septum with exfoliation of the vomer bone. (b) Approximately one-third of the lower middle nasal concha is removed on both sides. (c) The rear end of the membranous septa is removed on both sides. (d) The vomer bone is removed by drilling, and (e) the frontal cystic wall is resected with the pituitary scalpel. (f) Bones of the cranial base showed thinning, with pulsations and dura mater observed through them (black arrow). r IC, right internal carotid artery and l IC, left internal carotid artery. (g), (h) Cranial base reinforcement using fat and fascia collected from the femur, and (i) hemostatic packing.

For treatment, we performed a decompression of the mucocele using an endoscopic nasal approach (Figure 4). The cystic wall, which likely developed from the rear sphenoid sinus, showed significant thinning in a wide edge adjacent to the cranial base; therefore, we performed decompression of the frontal sphenoid sinus mucocele to the extent that was possible under general anesthesia. Although we did not identify cerebrospinal fluid leakage during surgery, we did observe pulsations in the broad osseous area of thinning at the cranial base. During the preoperative briefing, we had informed the patient that we would collect samples of the right fascia lata and fat in advance for a cranial base reinforcement if needed. We used these samples to cover the surface and to reinforce the cranial base.

**Figure 5:**
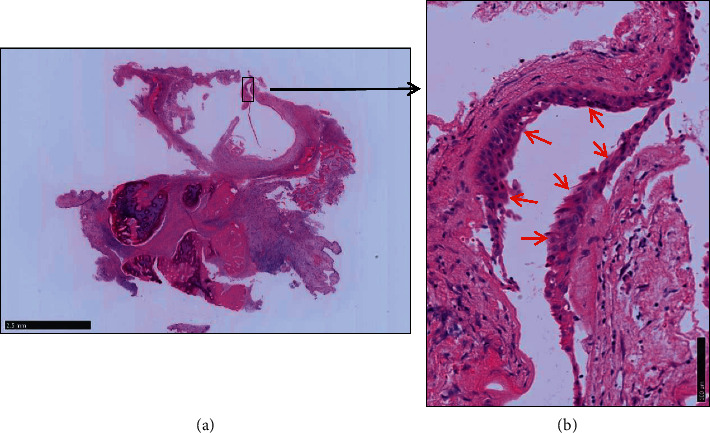
Hematoxylin and eosin staining. (a) Magnifying glass image and (b) 200× magnification. The cyst displays fibrous walls with squamous metaplasia of the ciliated pseudostratified epithelium due to chronic inflammation (red arrow).

**Figure 6:**
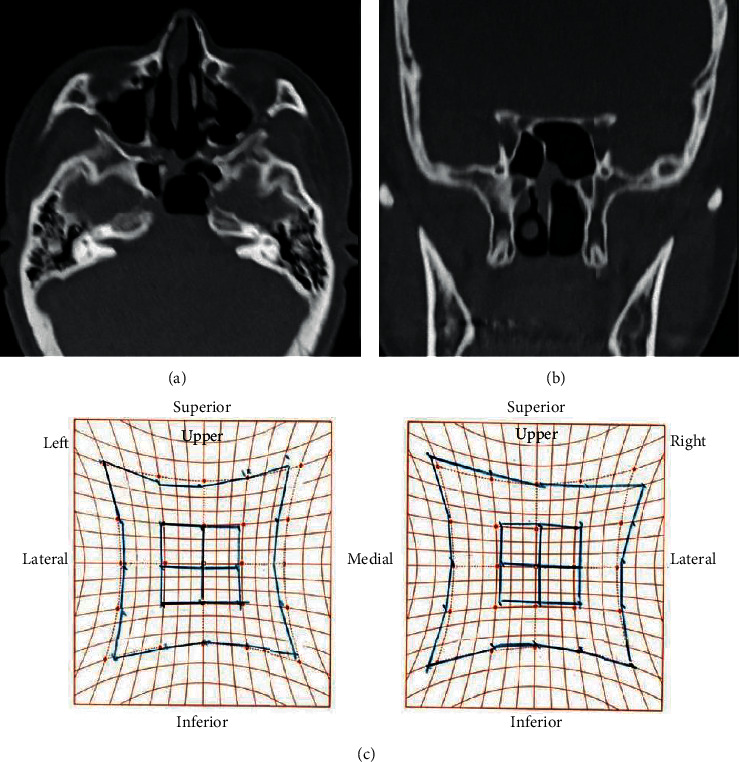
Postoperative evaluations. (a), (b) Postoperative computed tomography (plain) imaging of the paranasal cavity with a diminished sphenoid sinus mucocele, and (c) Hess screen test showing improvement in the abduction disturbance.

Diplopia began to subside immediately after surgery, with improvement in the electrooculography performed on the fifth day after surgery. Histopathology of the cystic wall showed a mucocele with fibrous walls, with squamous metaplasia of the ciliated pseudostratified epithelium in some portions due to chronic inflammation (Figure 5). As no arachnoid involvement was identified, the diagnosis was definitively determined to be a sphenoid sinus mucocele. No recurrence had occurred as of 30 months after the surgery (Figure 6).

## 3. Discussion

In this report, we presented a patient with an abducens nerve paralysis induced by a primary solitary sphenoid sinus mucocele with broad osseous thinning at the cranial base. We performed transnasal endoscopic surgery with a cranial base reinforcement using fascia lata and fat to avoid future cerebrospinal fluid leakage, with a successful outcome thus far.

Although this patient had no symptoms at presentation, headache is the most common presenting symptom in patients with sphenoid sinus mucoceles [6, 8], followed by ocular symptoms [6, 7, 9]. These patients often initially visit neurology departments for brain imaging or ophthalmology departments for eye evaluations. This can lead to a delay in diagnosis and can increase the possibility that some symptoms will remain after treatment. In this patient, lesions at the sphenoid sinus were incidentally identified by imaging performed during a complete medical checkup, leading to a relatively early diagnosis and quick referral to the ear, nose, and throat (ENT) surgery department.

The patient showed clivus loss at initial diagnosis, with thinning at the sella turcica and left internal carotid artery. While bones are relatively resistant to a sudden sporadic external force, they can be absorbed and thinned under conditions of continuous external force. Therefore, sphenoid sinus mucoceles are often associated with bone thinning at the cystic walls, resulting in fracturelike bone defects in the orbital bone and cranial base [5]. In these patients, surgical treatment is generally preferred for prevention of intracranial complications, regardless of the presence/absence of symptoms. In this case, we initially recommended close observation because the patient showed no symptoms at diagnosis and did not wish to undergo surgery at that time; however, a left abducens nerve paralysis developed 15 months after the initial diagnosis, prompting surgical management.

Approximately 94% of patients who report eye symptoms related to sphenoid sinus mucoceles report visual disturbances [6], with ocular motility disorders reported in 30–50% [7]. Often, both symptoms are reported concurrently by patients. A previous study reported that 70% of ocular motility disorders in these patients result from oculomotor paralysis, as the oculomotor nerve is more commonly affected by these lesions than the abducens or trochlear nerves [7]. However, multiple nerves can be anatomically disrupted in these cases, with a single nerve disturbance occurring more rarely [9]. In patients with sphenoid sinusitis, Lawson et al. reported that an abducens nerve paralysis can occur with optic nerve disturbances because the abducens nerve runs through the internal side of the cavernous sinus [10]. Hence, the cranial nerve responsible for motility disorders in these patients can differ based on the presence or absence of inflammation. In our case, we suspected several underlying mechanisms for the abducens nerve paralysis, including the mucocele's compression of the rear cavernous sinus, which is located at the lowest position relative to the nerves controlling the extraocular muscles, and the anatomical weakness of the abducens nerve, which may have been more vulnerable to pressure near the clivus due to its relatively long-running distance from the brainstem to the cavernous sinus [11]. The MRI findings did not show progression to Meckel's cave or Dorello's canal.

The most common surgical strategy for the management of sphenoid sinus mucoceles has now become drainage by a cystic wall puncture using a nasal endoscopic approach. Cranial base reinforcement with fat and fascia can also be considered in these patients. While a cranial base reinforcement can prevent fluid leakage after surgery, patients can also experience complications from harvesting of the fascia lata or fat. In our case, no cerebrospinal fluid leakage was noted; however, widespread bone thinning and pulsations at the cranial base were observed. Surgical trauma-induced cerebrospinal fluid leaks after endoscopic paranasal sinus surgery have been reported to occur in less than 0.1% of cases, with an even lower rate of delayed-onset cerebrospinal fluid leaks [12]. Idiopathic nasal cerebrospinal fluid rhinorrhea can also occur, but such cases are extremely rare [13, 14]. Although surgical treatment is commonly used for cerebrospinal fluid leaks, some cases spontaneously recover with a more conservative management strategy. While this lesion could have been treated without a cranial base fascial reinforcement and another surgery if necessary after the identification of a leak, we wanted to respect the patient's preference to avoid this future risk, as expressed in the preoperative briefing. Therefore, we performed drainage and cranial base reinforcement with fascia lata and fat, although without a nasal septum mucous membrane flap because of the lack of apparent cerebrospinal fluid leakage [13].

This report presents a patient with asymptomatic sphenoid sinus mucocele found incidentally during a complete medical checkup, leading to an early diagnosis and swift referral to the ENT department. Using a surgical approach, we were able to quickly address this lesion after the symptom manifested, resulting in a favorable outcome. This patient experienced no recurrence as of the last follow-up and likely has a favorable prognosis. In cases of sphenoid sinus mucocele, patients should be carefully evaluated and managed using either close observation or a surgical approach.

## Data Availability

The data used to support the findings of this study are available from the corresponding author upon request.
